# The Effect of Adding Job's Tears to Yogurt on Plasma Glycated Albumin, Weight, and Lipid Profile in Patients with Type 2 Diabetes Mellitus: A Randomized Controlled Trial

**DOI:** 10.1155/2022/1876731

**Published:** 2022-07-30

**Authors:** Nanny Djaja

**Affiliations:** Department of Public Health, School of Medicine, Atma Jaya Catholic University of Indonesia, Jakarta, Indonesia

## Abstract

**Background:**

A potential effect of Job's tears on metabolic diseases has been recognized. However, studies on the effect of Job's tears on lipid profile and glycated albumin (GA) are still rare. This study aimed to examine the influence of Job's tears in conjunction with probiotics on the lipid profile and GA concentration of patients with type 2 diabetes mellitus.

**Methods:**

This double-blind 12-week study involved 60 patients with type 2 diabetes assigned randomly into two groups. The first group consumed yogurt alone (containing *Lactobacillus acidophilus* La5 and *Bifidobacterium* Bb12), while the second had yogurt with Job's tears once daily (200 ml each). Lipids and GA concentrations were measured using an enzymatic colorimetric assay. Paired and unpaired Students *t*-test, Wilcoxon signed-rank, and Mann–Whitney test were applied. Statistical significance was set at *p* < 0.05.

**Results:**

The characteristics of the groups were comparable except for baseline plasma GA (*p*=0.012). Subjects who used Metformin were distributed equally between the groups (*p*=0.118). Caloric intake between the groups also did not differ (*p*=0.856). There was also no change in weight, BMI, or plasma GA. Yogurt and the mixture of Job's tears and yogurt reduced cholesterol and LDL and increased HDL (all *p* < 0.05) within the groups. However, HDL levels in patients who had Job's tears were significantly elevated than yogurt alone (0.9 vs. 25 mg/dL, *p*=0.029).

**Conclusion:**

The combination of Job's tears and yogurt improved HDL more than yogurt alone.

## 1. Introduction

The use of food containing probiotics for health purposes in humans has been increasing for decades. Yogurt is a probiotic enriched with lactic acid-producing bacteria such as *Lactobacilli* and *Bifidobacteria* [1]. These live microbiota improve intestinal flora balance, food absorption, intestinal mucosal integrity, body immunity, and resistance to infection [2, 3]. A study by Ejtahed et al. on type 2 diabetes mellitus (T2DM) showed that yogurt with *Lactobacillus acidophilus* La5 and *Bifidobacterium* Bb12 administered for 6 weeks significantly improved fasting plasma glucose (FBG), hemoglobin A_1C_ (HbA_1C_), and antioxidant enzyme activity [4]. The systematic review and meta-analysis of randomized controlled trial (RCT) studies by Ruan supports yogurt's effect on lowering plasma glucose [5]. Furthermore, yogurt consumption has also been shown to improve the lipid profile in T2DM [6, 7]. The use of herbal ingredients to treat diabetes has been known for a long time. Navebi et al. reported that *Melissa officinalis* L.-based products decrease triglyceride levels in diabetic patients [8]. Hashemi et al. described the beneficial effects of pomegranate seed powder on ih FBG and HbA_1C_ [9].

Job's tears (*Coix lacryma-jobi*), also known as adlay or adlay millet, is a grain from a plant of a grass family mainly found in Southeast Asia. People have recognized Job's tears' health benefits, and it is used as medicine for arthritis, malignancy, and hypercholesterolemia [10–12]. Job's tears' effects come from its ability to produce fructooligosaccharide (FOS), which functions as an antioxidant and stimulates certain indigenous gastrointestinal bacteria [8, 9]. The fermentation of FOS is accelerated by probiotic microbiota in the intestine to produce short-chain fatty acids (SCFAs; acetate, butyrate, propionate, etc.) [10]. Studies on animals and humans showed that SCFAs improve metabolism, insulin sensitivity, and adiposity [12, 13].

The American Diabetes Association (ADA) guidelines recommend HbA_1C_ as a diagnostic marker and treatment monitoring for T2DM besides plasma glucose concentration [14]. Besides its several advantages (requiring no fasting and stability), HbA_1C_ also has disadvantages, including lower sensitivity, higher cost, and less availability in developing countries [14]. Also, HbA_1C_ is influenced by hemoglobin metabolism and erythrocyte survival time, so it might not be recommended in T2DM with deficiency anemia, hemolytic anemia, hemoglobinopathy, chronic kidney disease, etc. [15]. Glycated albumin (GA) is a potential biomarker for diabetes because it is not influenced by the erythrocyte life span [15]. Relative to HbA1C, the concentration of albumin glycation is nine-fold higher, while the reaction speed is ten-fold faster [16].

Prebiotics added to probiotics (synbiotics) may enhance the beneficial effect of gut microbiota. The impact of adding Job's tears with yogurt on GA and lipid profiles on T2DM has not been investigated. Therefore, this study examines the effect of adding Job's tears to yogurt on plasma GA and lipid profiles in T2DM.

## 2. Methods

### 2.1. Subjects

This randomized controlled, parallel group trial was conducted from February to June 2018. The subjects were Atma Jaya employees with T2DM who regularly visited the employee clinic or those diagnosed with T2DM upon laboratory examination. Those with confirmed T2DM were referred to a specialist for appropriate treatment. The randomization process was conducted using balanced block methods. With a power of 90%, a sample size of 25 was obtained, plus a 25% dropout risk, resulting in 30 samples per group.

**Figure 1:**
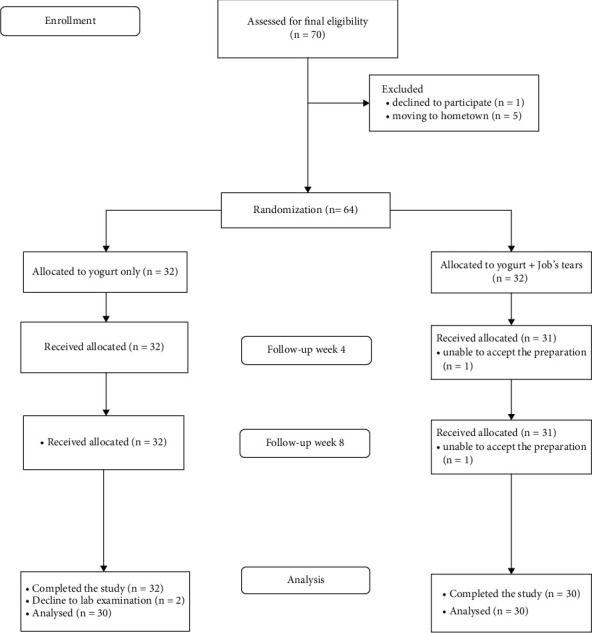
Standard CONSORT flowchart.

Subjects were then divided into two groups. The control group received yogurt only, and the intervention group received yogurt enriched with Job's tears. Subjects' allocation was balanced according to the oral antidiabetic used to avoid biasing changes in the measured variables. Sixty subjects (27 male and 33 female) met the following eligibility criteria: aged 30–60 years, T2DM, and not taking antidiabetic medicine or Metformin + 3 tabs/day (to minimize bias from the effect of maximal dose Metformin 3 tab/day). Criteria for T2DM were those with existing T2DM or fasting blood sugar (FBS) ≥ 126 mg/dL or random blood sugar (RBS) > 200 mg/dL measured in plasma. Exclusion criteria were: having BMI ≥ 30 kg/m^2^ (to ensure homogeny among subjects), taking antidiabetic medicine other than Metformin, antibiotic use in the last 6 weeks, consuming probiotic supplements in the previous 3 months, a history of cardiovascular disease, chronic liver disease, chronic kidney disease, malignancy, chronic diarrhea, chronic gastrointestinal disease, and pregnancy or breastfeeding. Subjects were randomly allocated into two groups, as described in Figure 1.

### 2.2. Ethical Considerations

All procedures were executed according to the Declaration of Helsinki. Information about the study was provided at least 7 days before the subjects signed the informed consent document. The Ethics Committee of the School of Medicine & Health Sciences, Atma Jaya Catholic University of Indonesia, approved the study (23/02/KEP-FKUAJ/2019).

### 2.3. Anthropometric Measurements

Height was measured at the Frankfort position using a stadiometer and is presented in cm approximated to the nearest 0.1 cm. Weight was measured using a digital scale (SECA, Robusta 813, Germany) in minimal clothing and is presented in kg to the nearest 0.1 kg. Body mass index (kg/m^2^) was calculated by dividing weight (kg) by height squared in meters. Subjects with a body mass index of less than 30 kg/m2 were included in this study. A dietician performed all measurements twice to get two similar results.

### 2.4. Food Intake Assessment

Food intake was assessed using 24-hour recall before and after the intervention. Food and beverages consumed were analyzed, including the type and amount of food, from midnight to midnight on the previous day. The amounts of food and drink were estimated according to household measures such as spoons, glasses, etc.

### 2.5. Blood Sample Analysis

Blood samples (3 ml) were obtained from the peripheral veins, were in a vacutainer, and then centrifuged at 1300–2000 rpm for 15 minutes to separate the serum using a standard device. The serum was collected in a tube and stored at −80°C before analysis. Glycated albumin concentration was measured using a colorimetric enzymatic method (Advia 1800 Chemistry System, Siemens Healthcare Diagnostics, Germany). The detection limit was 3.2%, and the measurement interval was 3.2–68.1%. Enzymatic colorimetric assay (Technicon Instruments, Ltd., New York, NY, USA) was used to determine serum total cholesterol and triglyceride concentrations. HDL cholesterol was determined enzymatically after precipitation of other lipoproteins in the supernatant with dextran sulfate magnesium. LDL cholesterol was calculated using the Friedewald formula [LDL = total cholesterol - (triglyceride/5)—HDL] [17].

### 2.6. Yogurt and Job's Tears Preparation

Fresh milk (1000 ml) was pasteurized at 70°C for 15 minutes. Some diluted skimmed milk was added. The milk was then stored in a pan and cooled at 45°C by putting it above the cold water. Cooled milk was then inoculated with lactic acid-producing bacteria, *Lactobacillus acidophilus La-5* and *Bifidobacterium Bb-12,* at 10^9^ CFU/ml each. Thereafter, the inoculated milk was insulated into an Erlenmeyer flask. The milk was fermented in an incubation device at 40–45°C for 4 hours. The pH was measured before and after fermentation to ensure the pH remained unchanged.

Job's tears extract was made from 250 g dry grains softened in water for 6 hours. Job's tears were then boiled in 1000 ml of water with Pandan leaves (*Pandanus amaryllifolius*) over medium heat (60°C) for 60 minutes and stirred every 15 minutes. Pandan leaves contain tannins, saponins, alkaloids, and flavonoids. A minimal amount of Pandan leaves was used for fragrance. The Job's tears were then cooled at room temperature (25°C) and filtered with Whatman filter paper no 1 (Whatman®, Germany). The filtrate was collected and concentrated using a rotary evaporator (R-124 Buchi, Switzerland). The final step was drying the filtrate with a lyophilizer (Alpha 1–2 LD; Christ, Germany).

The boiled Job's tears were blended into a porridge and mixed with yogurt before consumption. Both the yogurt and Job's tears-enriched yogurt preparations were stored in a refrigerator at 4°C.

### 2.7. The Administration of Yogurt and Job's Tears

Each subject was administered the same volume of the preparation, 200 ml of yogurt or 100 ml of yogurt with 100 ml of the Job's tears mixture. Both kinds of yogurt were placed in a sealed, opaque plastic cup and distributed by dieticians not involved in the study. Subjects consumed either yogurt or Job's tears-enriched yogurt once per day, 2 hours after lunch for 12 weeks while being monitored by a dietician (who was not involved in the study). The preparations were provided in the office during weekdays. Subjects picked up the preparations for the weekend on Fridays. Subjects were advised to store the preparations in a refrigerator to keep them cool. Consuming the preparation on the weekend was self-monitored using the video camera on the subjects' cellphone.

Double blinding was applied in this study. The preparations had the same color and the same aroma which could not be distinguished by the patients. The dieticians who distributed the preparation did not know the kind of preparation.

### 2.8. Statistical Analysis

Numerical data were presented as means ± standard deviations and categorical data as frequency. The normality of the data distribution was tested with the Shapiro–Wilk's test. The intervention effects within the group were evaluated using the paired sample *t*-test or Wilcoxon signed-rank test. The effects between the groups were analyzed using the independent samples *t*-test or Mann–Whitney test. Chi-square was applied to assess the distribution of Metformin use among the groups. All statistical analyses were computed using SPSS version 19. Statistical significance was set at *p* < 0.05.

## 3. Results

Seventy participants were originally eligible for the study, but six participants withdrew (one declined and five moved to their hometown). The remaining 64 participants were randomized—32 per group. After 4 weeks of follow-up, one participant receiving the Jobs' tears and yogurt mixture dropped out due to stomach discomfort, and at the eighth-week follow-up, another participant from that same group also dropped out due to stomach discomfort after consuming the preparation. Two participants receiving yogurt alone declined the laboratory examination. Thus, 30 participants were included in each group for the final analysis (Figure 1).

The preparation was not well tolerated by a few participants, and two of them decided to withdraw. The minor adverse events reported by six participants included diarrhea by two participants (once each), heartburn, bloated stomach, stomach discomfort, and softer stool experienced by each one (once each) in which two of them were withdrawn.

Table 1 compares the characteristics between the groups. Age, anthropometric measures (height, weight, and BMI), and calorie intake did not differ significantly between the groups (all *p* > 0.05). The distribution of subjects with medication (Metformin) was balanced between the groups (*p*=0.374). Plasma GA was significantly higher in the yogurt alone group than in the mixture of yogurt and Job's tears group (*p*=0.012).

The intervention effects on weight, BMI, and serum GA within the groups are described in Table 2. Within the yogurt alone group, except for plasma GA (*p*=0.356), all variables significantly changed (*p* < 0.05). Within the Job's tears-enriched yogurt group, all variables changed significantly, including a decrease in plasma GA (*p*=0.022). As observed in the yogurt alone group, TG increased significantly (*p*=0.022).

The effect of the intervention between the groups was compared. The changes (Δ) in all variables, except for HDL, were not significant. HDL increased significantly after the mixture of yogurt and Job's tears compared with yogurt alone (*p* < 0.029). Although not significant, the mixture of yogurt and Job's tears reduced plasma GA levels (−0.2%) (Table 3).

## 4. Discussion

The effects of prebiotics and probiotics on health have been widely investigated. However, studies involving human intervention are few. Most studies on T2DM focus on the effects of prebiotics and probiotics on plasma glucose. Nevertheless, diabetes is often accompanied by dyslipidemia, and a more reliable improvement biomarker of diabetes is needed. This study examined yogurt's effect compared with Job's tears-enriched yogurt administration on the lipid profiles and glycated plasma albumin levels in diabetic patients. This study found that yogurt and Job's tears-enriched yogurt reduced weight and BMI and improved lipid profile (total cholesterol, LDL, and HDL). Compared with yogurt alone, Job's tears-enriched yogurt significantly changed HDL levels only.

Many studies on the effect of probiotics on cholesterol and triglyceride levels have been conducted. However, the results vary considerably between studies. An RCT by Tonucci et al. [18] found a significant decrease in total cholesterol and LDL in T2DM after consuming probiotics containing *Lactobacillus* La5 and *Bifidobacterium lactis* Bb12 (10^9^ cfu/d, each) for 6 weeks [18]. A similar finding was reported by four meta-analysis studies [19–22]. Total cholesterol, LDL, and TG were significantly reduced with probiotic supplementation [19–22]. Several studies also reported different results. An RCT study by Sabico et al. [23] investigated the effects of 6-month probiotic supplementation in T2DM patients. The results indicated that total cholesterol, LDL, HDL, and TG did not change significantly. This result was supported by Ivey et al. [24], who reported that the lipid profile was not affected by a 6-week probiotic supplementation. Our study indicated that lipids were improved in T2DM after probiotic and synbiotic supplementation for 12 weeks. However, only HDL was significantly reduced after Job's tears-enriched yogurt administration compared with yogurt alone. The discrepancy in the findings might be attributed to the subjects' characteristics, types, and administration duration. Unfortunately, TG significantly increased in both the groups. The cause remains unclear but might relate to participants' food consumption, which was not controlled or determined.

Many mechanisms for the cholesterol-lowering effects of probiotics have been proposed. When probiotics are present in the intestine, they ferment indigestible carbohydrates to produce SCFAs [25]. SCFAs can lower lipids by blocking the synthesis of hepatic cholesterol. Probiotics also deconjugate bile acids to become bile salts through an enzymatic process [26]. The cholesterol-lowering effect of probiotics is also attributed to their ability to remove cholesterol by binding to small intestine cells' surfaces [27, 28]. In contrast, the effect of herbal ingredients on improving blood sugar is likely mediated by *β*-cell function and *β*-cell regeneration via the reduction of oxidative stress and inflammation [29].

Combining prebiotics and probiotics enhances their singular effects. A combination of prebiotics and probiotics (synbiotic) produces a synergistic effect that can enhance the treatment capacity. A study on the combined effect of probiotics and prebiotics in healthy women showed that the combination of probiotics and prebiotics significantly increased the proportion of Bacteroides in stool [30]. Also, synbiotics were more effective compared with prebiotics and probiotics alone in reducing C-reactive protein and improving social and systemic function in ulcerative colitis [31]. A study by Rajkumar et al. [32] examined the efficacy of combining prebiotics and probiotics. The synbiotic (FOS with *L. salivarius*) was more beneficial in lowering lipids than the probiotic alone (*L salivarius*) in healthy young people [32]. Efficacy will also increase if the probiotics contain multiple species. A meta-analysis study by Nikbath et al. [33] found that multispecies probiotics have a more significant effect than single-species preparations in reducing plasma glucose. A study by Zhang et al. [34] confirmed that multiple species of probiotics can reduce weight and BMI. The recent study used probiotics containing the most abundant microbiota—*Lactobacillus acidophilus* La5 and *Bifidobacterium* Bb12. Our findings showed a significant improvement in serum HDL and GA after synbiotic (yogurt and Job's tears) administration compared with a probiotic (yogurt) alone.

Hyperglycemia and dyslipidemia accelerate the production of glycation end products [35]. Thus, GA (albumin bound to glucose) will also increase under conditions of hyperglycemia and dyslipidemia. Glycated albumin seems to be a better biomarker than HbA_1C_ in cases of diabetes mellitus [15, 16, 36]. Nevertheless, studies on the effect of diabetes treatment on serum GA are still scarce. One study investigated the effect of probiotic supplementation on advanced glycation in adults with prediabetes [37]. Glycation end product was used as a biomarker, and the results showed that it was reduced after prebiotic supplementation. This recent study might be the first to examine serum GA changes due to synbiotic administration. Our study demonstrated that a mixture of yogurt and Job's tears effectively reduced plasma GA. Reduced plasma GA was not found after the administration of yogurt alone. These findings indicate that reduced plasma GA might be attributed more to increased HDL in connection with dyslipidemia.

This study has some limitations. First is the lack of controlling the subjects' diet during the study. Controlling eating behavior is essential to ensure the subjects consumed the supplement and no food source that may interfere with the results. Second, physical exercise may also create bias due to its effect on plasma glucose. However, we only noted a few subjects engaged in physical exercise 1–2 days/week, which likely has little to no effect on plasma glucose changes.

## 5. Conclusion

This study investigated the effect of adding Job's tears (*Coix lacryma-jobi*) containing FOS on yogurt in improving carbohydrate and lipid metabolism in people with T2DM and body size. The parameters of CH metabolism (plasma glucose, plasma GA), lipid metabolism (lipid profile), and body size (weight, BMI) were evaluated. The significant effect of adding Job's tears on yogurt was an increase in HDL. However, the results should be interpreted with caution due to several limitations.

## Figures and Tables

**Table 1:** Characteristics of subjects.

	Yogurt (*n* = 30)	Yogurt with Job's tears (*n* = 30)	*p*
Gender			
Male	14	13	—
Female	16	17	
Age (years)	45.7 ± 7.8	43.4 ± 9.2	0.312
Height (cm)	160.7 ± 7.2	158.5 ± 6.5	0.208
Weight (kg)	64.7 ± 9.9	65.9 ± 10.2	0.617
BMI (kg/m^2^)	24.9 ± 2.7	26.4 ± 4.6	0.145
Food calorie intake (kcal)	1928.8 ± 146.8	1944.2 ± 127.5	0.856
Total cholesterol (mg/dL)	178.8 ± 26.7	183.2 ± 25.9	0.528
LDL (mg/dL)	132.8 ± 27.2	137.1 ± 26.2	0.542
HDL (mg/dL)	45.0 (40–49)	45.0 (42–50)	0.688^*∗*^
Triglycerides (mg/dL)	126.5 (43–285)	125.5 (41–305)	0.638^*∗*^
Plasma GA (%)	19.3 (11.3–33.1)	13.6 (10.7–49)	0.012^*∗*^

Medication			
No medication	10	16	0.118
Metformin	20	14	

^*∗*^Mann–Whitney *U* test. Abbreviations: BMI, body mass index; GA, glycated albumin; LDL, low-density lipoprotein; HDL, high-density lipoprotein.

**Table 2:** Effect of intervention within the groups.

	Yogurt (*n* = 30)			Yogurt + Job's tears (*n* = 30)		
	Pre	Post	*p*	Pre	Post	*p*
Weight (kg)	64.7 ± 9.9	63.6 ± 9.3	≤0.001	65.9 ± 10.2	64.6 ± 9.5	≤0.001
BMI (kg/m^2^)	24.9 ± 2.7	24.5 ± 2.6	0.004	26.4 ± 4.6	25.8 ± 4.4	0.002
Total cholesterol (mg/dL)	178.8 ± 26.7	173.1 ± 21.0	0.015	183.2 ± 25.8	173.7 ± 23.8	≤0.001
LDL (mg/dL)	132.8 ± 27.2	126.0 ± 21.7	0.004	137.1 ± 26.2	125.7 ± 24.1	≤0.001
HDL (mg/dL)	45.0 (40–49)	47.1 (37–56)	0.015^*∗*^	45.0 (42–50)	48.0 (42–58)	0.002^*∗*^
Triglycerides (mg/dL)	126.5 (43–285)	178.0 (90–296)	0.001^*∗*^	125.5 (41–305)	184.5 (105–270)	0.022^*∗*^
Plasma GA (%)	19.3 (11.3–33.1)	18.5 (11–30.8)	0.356^*∗*^	13.6 (10.7–49)	13.0 (10.8–51.8)	0.22^*∗*^

^*∗*^Wilcoxon signed-rank test. Abbreviation: BMI, body mass index; GA, glycated albumin; LDL, low-density lipoprotein; HDL, high-density lipoprotein.

**Table 3:** The effects of intervention between the groups.

Variables change	Yogurt (*n* = 30)	Yogurt + Job's tears (*n* = 30)	*p*
^Δ^Weight	−1.1 ± 1.1	−1.3 ± 1.3	0.351
^Δ^BMI	−0.4 ± 0.4	−0.5 ± 0.5	0.294
^Δ^Total cholesterol	−5.0 ((−24)−26)	−9.0 ((−38)−12)	0.311^*∗*^
^Δ^LDL	−6.5 ((−27)−24.8)	−12.0 ((−114)−9)	0.105^*∗*^
^Δ^HDL	1.0 ((−8)−11)	2.9 ((−5)−10)	0.029^*∗*^
^Δ^Triglycerides	46.6 ± 68.6	29.7 ± 57.3	0.312
^Δ^Plasma GA	−0.2 ((−3.9)−6.1)	−0.4 ((−3)−7.4)	0.559^*∗*^

^*∗*^Mann–Whitney *U* test. Abbreviation: BMI, body mass index; LDL, low-density lipoprotein; HDL, high-density lipoprotein; GA, glycated albumin.

## Data Availability

The data used to support the findings of the study are available at https://drive.google.com/drive/folders/1S_Gkd4V3pAOkjbVtGlHF7btu34iqHWZZ?usp=sharing.
